# An aggregation inhibitor specific to oligomeric intermediates of Aβ42 derived from phage display libraries of stable, small proteins

**DOI:** 10.1073/pnas.2121966119

**Published:** 2022-05-17

**Authors:** Sara Linse, Pietro Sormanni, David J. O’Connell

**Affiliations:** ^a^Biochemistry and Structural Biology, Lund University, SE-22100 Lund, Sweden;; ^b^Chemistry of Health, Yusuf Hamied Department of Chemistry, Cambridge University, Cambridge CB2 1EW, UK;; ^c^School of Biomolecular and Biomedical Science, University College Dublin, Dublin D04 V1W8, Ireland;; ^d^BiOrbic, Bioeconomy SFI Research Centre, University College Dublin, Dublin 04 V1W8, Ireland

**Keywords:** self-assembly, affinity selection, reaction intermediates, novel scaffold, binder

## Abstract

The self-assembly of amyloid β peptide (Aβ) to fibrillar and oligomeric aggregates is linked to Alzheimer’s disease. Aβ binders may serve as inhibitors of aggregation to prevent the generation of neurotoxic species and for the detection of Aβ species. A particular challenge involves finding binders to on-pathway oligomers given their transient nature. Here we construct two phage–display libraries built on the highly inert and stable protein scaffold S100G, one containing a six-residue variable surface patch and one harboring a seven-residue variable loop insertion. Monomers and fibrils of Aβ40 and Aβ42 were separately coupled to silica nanoparticles, using a coupling strategy leading to the presence of oligomers on the monomer beads, and they were used in three rounds of affinity selection. Next-generation sequencing revealed sequence clusters and candidate binding proteins (SXkmers). Two SXkmers were expressed as soluble proteins and tested in terms of aggregation inhibition via thioflavin T fluorescence. We identified an SXkmer with loop–insertion YLTIRLM as an inhibitor of the secondary nucleation of Aβ42 and binding analyses using surface plasmon resonance technology, Förster resonance energy transfer, and microfluidics diffusional sizing imply an interaction with intermediate oligomeric species. A linear peptide with the YLTIRLM sequence was found inhibitory but at a lower potency than the more constrained SXkmer loop. We identified an SXkmer with side-patch VI-WI-DD as an inhibitor of Aβ40 aggregation. Remarkably, our data imply that SXkmer-YLTIRLM blocks secondary nucleation through an interaction with oligomeric intermediates in solution or at the fibril surface, which is a unique inhibitory mechanism for a library-derived inhibitor.

## Significance

Alzheimer’s disease affects a growing number of people, but a cure is lacking. The disease is connected to the formation of plaques in the brain, the first of which appear years before the first symptoms. Current approaches fail to stop or revert the propagation of these plaques, which are also a source of neurotoxic species in the form of oligomers. This work represents two directions toward therapeutic developments: 1) the design and production of protein libraries based on a small and stable scaffold, and 2) the realization of a screening procedure that allows for the identification of oligomer binders. The approach is successful in identifying a candidate protein that binds to oligomers and reduces the rate of plaque proliferation.

Alzheimer’s disease (AD) is a devastating neurodegenerative disease for which there is currently no cure (1, 2). The pathology of AD has been linked to the aggregation of two proteins: amyloid β peptide (Aβ) and the protein tau (3). Both proteins are found to exist as monomers, oligomers of various sizes, some of which are referred to as protofibrils, as well as highly ordered amyloid fibrils (4–7). According to the current consensus in the field, monomers and fibrils have relatively low toxicity to neurons compared to oligomeric intermediates. However, the aggregation process of Aβ is autocatalytic, and a majority of new oligomers are generated from monomers on the surface of existing fibrils (8). The generation of new aggregates is dominated by secondary nucleation on the side of fibrils, which has a much lower energy barrier than primary nucleation (9). Oligomers are transient species that may either convert to fibrils or dissociate into monomers that may undergo new cycles of oligomer formation and conversion (10).

High-affinity binders for Aβ monomers and aggregates have distinct effects on the aggregation process and the evolution of toxicity (11). Inhibitors of secondary nucleation may limit the concentration of oligomers during the reaction and the toxicity caused to neurons in brain tissue (12–14). Inhibitors of primary nucleation may instead delay the onset of aggregation, and although the generation of toxicity is not reduced it may be greatly delayed (15). Binding proteins for aggregates as well as monomers may thus provide valuable tools for therapeutics and diagnostics.

**Fig. 1.**
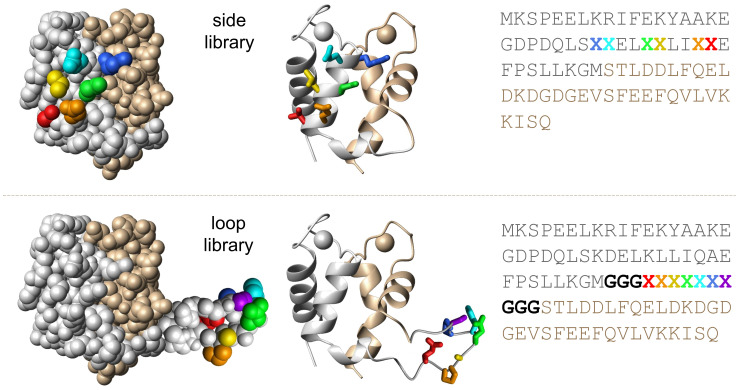
SXkmer library design. The SXkmer side library (*Top*) includes a six-residue randomized patch at the side of EF1 (5·10^7^ theoretical diversity). The loop library (*Bottom*) includes a 13-residue insert with three glycine residues (bold) at either side of a seven-residue randomized segment inserted between EF1 and EF2 (9·10^8^ theoretical diversity). The crystal structure of calbindin D_9k_ (3icb.pdb), is shown as space-filling models (*Left*) and ribbon diagrams (*Right*) with EF1 shown in silver and EF2 in gold. The backbone of the loop inserted between residues 43 and 44 is shown in white. The side chains that are randomized over 19 residue types, excluding cysteine, are shown in purple, blue, cyan, green, yellow, orange, and red in the structural models and are indicated by X in the sequences.

There is an urgent need for improved tools and scaffolds for inhibitor development. In the present study we therefore produced two phage–display libraries for the selection of binding proteins using human S100G, also known as calbindin D_9k_, as a scaffold. S100G is a small and tightly folded single-domain protein composed of two EF-hands [EF1 and EF2; Fig. 1 (16)]. The choice of S100G as a scaffold was motivated by its remarkable stability, as shown by its resistance toward proteolysis and denaturation. With bound Ca^2+^, the protein does not denature even when boiled in 8 M urea (17). Another desirable feature is its remarkable inertness to protein–protein interaction. S100G is a highly soluble protein with a highly hydrophilic surface, which has been employed as a negative control in protein array screening studies (18). The protein can be reconstituted with high affinity from its EF-hands (19) and is highly tolerant to loop insertions between the EF-hands (20). S100G is therefore an attractive scaffold for building target recognition functionality.

Two libraries were constructed in the form of a side library with a structurally constrained six-residue variable patch on the surface of EF1 and a loop library with a seven-residue variable loop insertion between EF1 and EF2 (Fig. 1), which we termed SXkmer libraries. The size of the potential binding surface and its theoretical diversity of ∼5 × 10^7^ and ∼9 × 10^8^ for the side and loop libraries, respectively, should be sufficient to yield binders for a variety of targets, including amyloidogenic proteins such as the Aβ peptide. In practice, higher theoretical diversity often leads to incomplete libraries given that limitations in transformation efficiency and propagation constrain the achievable diversity in library production and screening. Furthermore, for the side library, randomizing a bigger surface area could lead to a destabilization of the S100G scaffold, meaning that a portion of the resulting library would be poorly stable, which is undesirable for binder selection. While the dimensions of the folded part of Aβ42 fibrils is ca. 2.5 × 2.5 nm per monomer (5) with four monomers per plane (21), peptides of 5 to 9 residues have been shown to form stable amyloid fibrils (22) and previous studies have reported highly selective amyloid-binding peptides or antibody loop regions of 7 to 8 amino acid residues length (23–25), further supporting our choice of 6 or 7 varied residues in the libraries.

The two libraries were used to select binders for Aβ40 and Aβ42 in the form of monomers/oligomers and fibrils. In the latter case, fibril binders were enriched after the depletion of monomer/oligomer binders in an attempt to derive conformation-specific binders in accordance with previous work (13). The eluates after three rounds of selection were subjected to next-generation sequencing (NGS) for the identification of prominent sequences and sequence clusters, followed by the expression and purification of candidates as free SXkmer proteins. Their activity as aggregation inhibitors was assayed using a thioflavin T (ThT) fluorescence using nonseeded and seeded samples, and the interaction with Aβ was further analyzed using surface plasmon resonance (SPR) technology, Förster resonance energy transfer (FRET), and microfluidics diffusional sizing (MDS).

## Results

### Library design and characterization.

We designed two SXkmer libraries for phage display. The first library, called the side library, contains 6 randomized positions on the side of the EF1 domain, which are positioned nearby in the 3-dimensional space to enable the formation of a binding surface (Fig. 1). The second library, called the loop library, contains a loop of 13 residues inserted between the EF1 and EF2 subdomains. This inserted loop consists of 3 glycine residues per side to deliver flexibility that flank a randomized motif of 7 residues (Fig. 1). In both libraries, the randomized positions are uniformly sampled among 19 natural amino acids; cysteines were excluded because solvent-exposed cysteines can promote dimerization and are typically regarded as liabilities. The DNA design also excluded all stop codons except the Amber stop codon TAG, which codes for Gln in the *Escherichia coli* host Tg1 used for the phage library production and amplification. The exact codon usage is shown in *SI Appendix*, Table S1. Consequently, the side library has a theoretical diversity of 19^6^ ∼ 5·10^7^ and the loop library has a theoretical diversity of 19^7^ ∼ 9·10^8^.

The libraries were generated through DNA synthesis and cloning into the pIT2 phagemid vector. The quality of these naïve libraries was verified by NGS sequencing before any screening. Almost 10^6^ reads were sequenced from each library (specifically, 818,985 and 637,624 reads for the side and loop libraries, respectively; *SI Appendix*, Fig. S1). An analysis of these reads revealed that for both libraries approximately 80% of reads corresponded to unique sequences, and more than 99.9% corresponded to sequences observed no more than twice (*SI Appendix*, Fig. S2*A*). This result suggests that the diversity of the libraries is high and, more important, it indicates that there are no biases, as no motif was found to be substantially enriched before screening. An analysis of amino acid frequency at the randomized positions further revealed only small deviations from the expected uniform distribution, as one may expect from the rather limited sampling of the theoretical diversity (*SI Appendix*, Fig. S2*B*). Taken together, these results show that the final libraries are in full agreement with the intended design and that there is plenty of diversity for the successful screening and selection of binders.

### Antigen immobilization and selection of binders.

The two SXkmer libraries, loop and side (Fig. 1), were used in three rounds of selection with an aim to retrieve both monomer and fibril binders to Aβ40 as well as Aβ42. Toward this aim, recombinantly expressed and purified monomers of Aβ40 and Aβ42 were separately coupled to silica nanoparticles. Monomer samples were also used to generate Aβ40 and Aβ42 fibrils, as validated using ThT fluorescence, which were separately coupled to silica nanoparticles. We note that the coupling of monomers to the nanoparticles at acidic pH may result in a mixture of monomers and oligomers. The selection of monomer and oligomer binders used the monomer beads only. The selection of fibril binders was preceded by the depletion of monomer and oligomer binders on monomer-coupled beads before incubation with fibril-coupled beads.

### NGS of eluates.

**Fig. 2.**
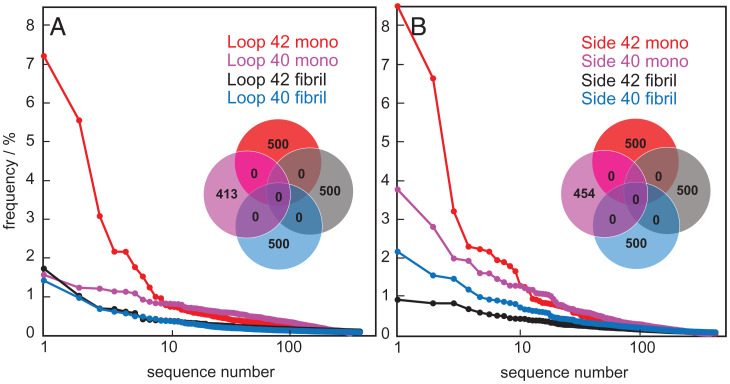
The frequency of occurrence of the top 500 hits from the third round of selection of (*A*) loop library and (*B*) side library versus Aβ42 monomer, Aβ40 monomer, Aβ42 fibril, and Aβ40 fibril. Distribution of the top 500 sequences with at least 100 reads each in each selection shown with Venn diagrams.

**Fig. 3.**
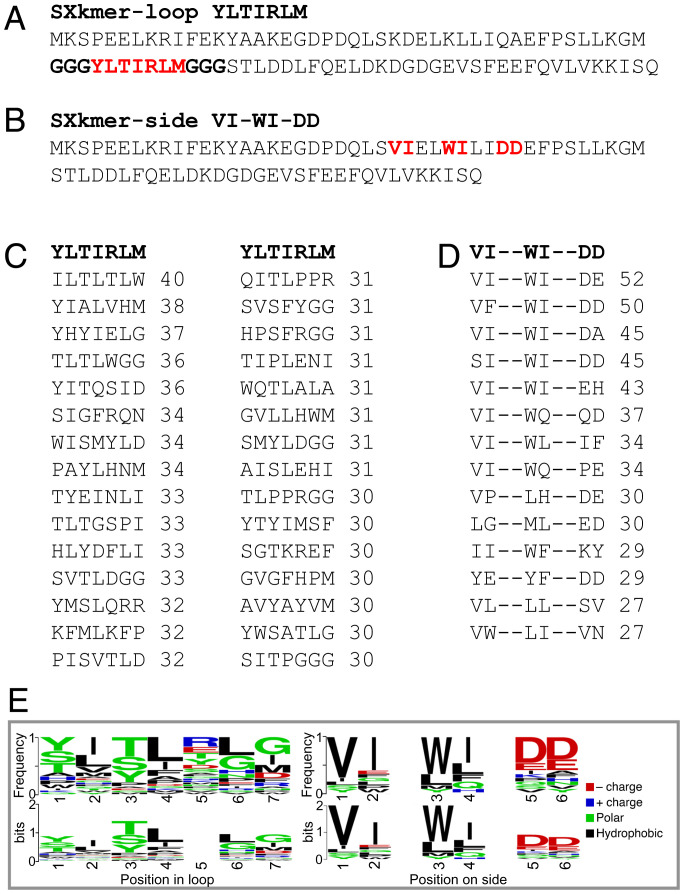
Amino acid sequences of the two SXkmers produced in this work as binders of (*A*) Aβ42 monomer or oligomer, SXkmer-YLTIRLM, and (*B*) Aβ40 monomer or oligomer, SXkmer-VI-WI-DD. Bold red letters are used to indicate the amino acid residues appearing at the positions that were randomized in the respective library (see Fig. 1). Bold black letters show the three glycine residues introduced at each side of the loop insert. (*C* and *D*) Alignment clusters showing all sequences with a score of at least 30 versus YLTIRLM (*C*) or 27 versus VI-WI-DD (*D*, side library). The score for 100% identity would be 63 (loop library) or 56 (side library). (*E*) Frequency (*Top*) and bits (*Bottom*) sequence logos (26) of the sequences identified in the screening, which are similar to loop YLTIRLM (*Left*) and side-patch VI-WI-DD (*Right*). In the bits logo, the overall height of the stack indicates the sequence conservation at that position, while the height of symbols within the stack indicates the relative frequency of each amino acid at that position. Logos were drawn with the WebLogo tool (26).

The eluates after three rounds of selection were subjected to PCR using primers inside the constant part of S100G spanning the variable regions followed by NGS to provide information on the retrieved side and loop sequences. The frequency of the most abundant sequence and the number of sequences that occurred at least 100 times (i.e., with at least 100 reads) after three rounds of selection are listed in Table 1. The frequency of occurrence of the top 500 sequences from each selection were compared, and the sequence distribution between the third-round selections are shown in the form of Venn diagrams (Fig. 2). The sequences of the top 10 hits in each case are listed in *SI Appendix*, Table S2. The sequences of two investigated hits with alignment clusters of similar sequences are shown in Fig. 3. Further analysis of the YLTIRLM sequence cluster using the Weblogo tool (26), reveals a pattern of alternating hydrophilic and hydrophobic residues (Fig. 3*E*). Positions 2, 4, and 6 show a preference for hydrophobic residues, in particular leucine or isoleucine, while positions 1, 3, and 5 are occupied by polar residues and tend to be more varied. Positions 1 and 3 are predominantly occupied by Y, S, or T. Position 5 is poorly conserved and seems able to tolerate negative, positive, or polar residues, but notably not hydrophobic ones. The last position, 7, appears to be outside of the alternating pattern as it allows both polar and hydrophobic amino acids. For the side-patch VI-WI-DD cluster, a rather strong conservation is observed at all six positions; the first four positions are occupied preferentially by large hydrophobic residues and the last two by negatively charged residues.

**Table 1.** Summary of the eight screens for Aβ binders, showing the highest frequency of occurrence of any sequence (Top freq) and the number of sequences found at least 100 times (N seq) in the NGS data after three rounds of selection (5.10^6^ reads per selection)

Selection	Library	Target	Depleted on	Top freq (%)	N seq
Loop40mono	S100GLoop	Aβ40 monomer	—	1.5	417
Loop40fib	S100GLoop	Aβ40 fibril	Aβ40 monomer	1.4	6,212
Loop42mono	S100GLoop	Aβ42 monomer	—	7.2	661
Loop42fib	S100GLoop	Aβ42 fibril	Aβ42 monomer	1.7	3,082
Side40mono	S100GSide	Aβ40 monomer	—	3.7	458
Side40fib	S100GSide	Aβ40 fibril	Aβ40 monomer	2.1	2,483
Side42mono	S100GSide	Aβ42 monomer	—	8.5	690
Side42fib	S100GSide	Aβ42 fibril	Aβ42 monomer	0.9	3,874

### Production of candidate SXkmers.

The loop sequence YLTIRLM and the side-patch VI-WI-DD were selected based on their frequency of occurrence and the presence of many homologous sequences for both (Fig. 3) based on sequence analysis (see *Methods*). These two sequences were reintroduced into human S100G and expressed as free SXkmer proteins. The proteins were found to be expressed at very high yield of approximately 1 g/L (SXkmer-YLTIRLM) and 200 mg/L (SXkmer-VI-WI-DD) and were purified using sonication; boiling; two anion exchange steps in EDTA and Ca^2+^, respectively; and size exclusion chromatography (see *Methods* and *SI Appendix*, Fig. S3). The full amino acid sequences of these two proteins are shown in Fig. 3.

### Aggregation kinetics of Aβ42.

**Fig. 4.**
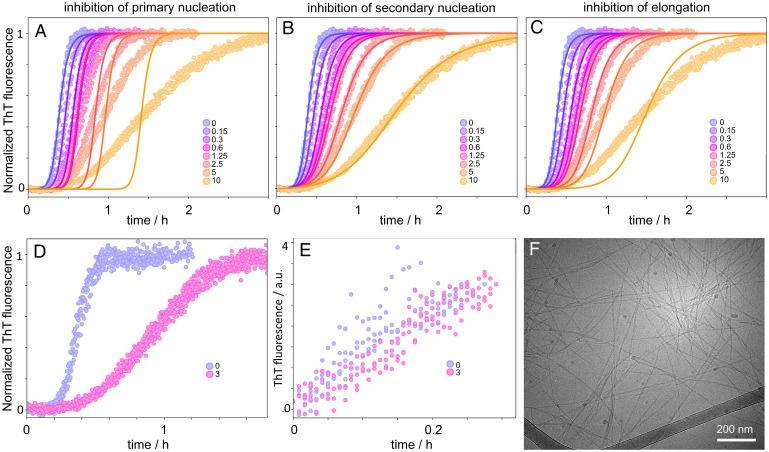
Effect of SXkmer-YLTIRLM on Aβ42 aggregation kinetics. (*A*–*C*) Aggregation kinetics of 3 µM Aβ42 in the absence and presence of SXkmer-YLTIRLM (separate colors for each concentration, as indicated in the legend in µM) in 10 mM Tris, 150 mM NaCl, and 100 µM CaCl_2_, pH 8.0 monitored by ThT fluorescence. The data were fitted three times, assuming a selective reduction of the rate constant for primary nucleation (*A*), secondary nucleation (*B*), and elongation (*C*). (*D* and *E*) Seeded aggregation. Data obtained without (purple) and with (pink) 3 µM SXkmer-YLTIRLM for reactions starting from 2 µM Aβ42 monomer supplemented with 1% seed (*D*) or 30% seed (*E*, a.u., arbitrary units). (*F*) Cryo-transmission electron microscopy (cryo-TEM) image of Aβ42 fibrils formed in the presence of SXkmer-YLTIRLM. Additional cryo-TEM images of fibrils formed from Aβ42 in the presence of SXkmer-YLTIRLM as well as Aβ42 alone are shown in *SI Appendix*, Fig. S4.

The effect of SXkmer-YLTIRLM on the aggregation kinetics of Aβ42 was monitored using ThT fluorescence (Fig. 4). The data were analyzed using the Amylofit platform (27), and three modes of fitting were tested assuming the selective reduction of the rate constant for k_n_, k_2_, and elongation (k_+_). Clearly, the model assuming the selective reduction of k_2_ fit the data best, indicating that YLTIRLM selectively inhibits secondary nucleation.

To confirm the results of the fitting of the unseeded reaction, seeded experiments were carried out in different conditions. First, the aggregation starting from monomers was supplemented with a low concentration (1%) of seed fibrils. Under these conditions, the slower primary nucleation is bypassed, and secondary nucleation dominates the reaction (8). As expected, the results of this assay clearly showed that SXkmer-YLTIRLM inhibits the aggregation, confirming its effect on the secondary pathways (Fig. 4*D*). Second, a similar experiment was carried out at a high seed concentration (30% seed). At these heavily seeded conditions, the dominant process is fibril elongation (9). As expected from the fit of the unseeded reaction, SXkmer-YLTIRLM had very little inhibitory effect on the heavily seeded assay, thus confirming its lack of effect on the elongation process (Fig. 4*E*). Taken together, these results show that SXkmer-YLTIRLM blocks secondary nucleation but does not interfere with elongation of the seed fibrils (Fig. 4).

**Fig. 5.**
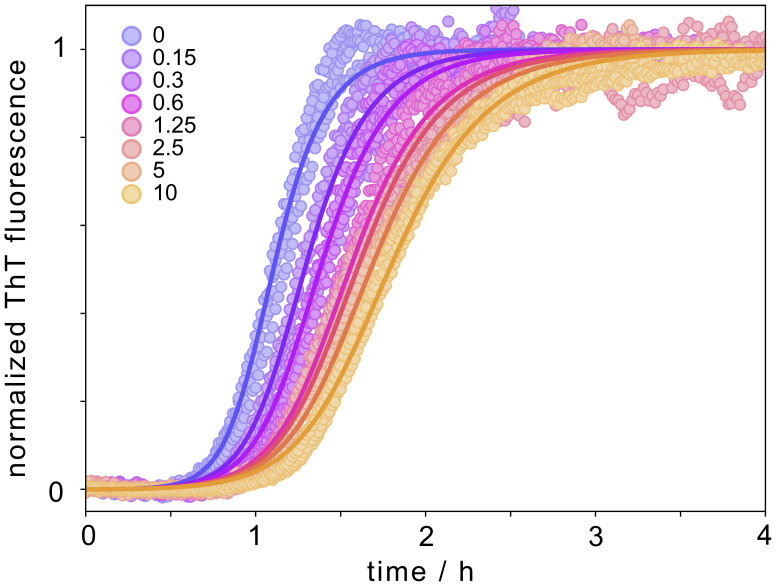
Effect of synthetic peptide YLTIRLM on Aβ42 aggregation kinetics. Aggregation kinetics of 3 µM Aβ42 in the absence and presence of YLTIRLM (separate colors for each concentration as indicated in µM) in 20 mM sodium phosphate, 0.2 mM EDTA, pH 8.0 monitored by ThT fluorescence. The data were fitted assuming the selective reduction of the rate constant for secondary nucleation. Additional fits are shown in *SI Appendix*, Fig. S5.

A synthetic peptide YLTIRLM was prepared and was found to inhibit secondary nucleation with a lower potency but in a similar manner to the constrained sequence in the SXkmer protein (Fig. 5).

### Aggregation kinetics of Aβ40.

The effect of SXkmer-VI-WI-DD, derived from the side library on the Aβ40 aggregation kinetics was monitored by ThT fluorescence (*SI Appendix*, Fig. S6). The data were analyzed using the Amylofit platform. Three modes of fitting were tested assuming selective reduction of the rate constant for k_n_, k_2_, and k_+_. Here, the distinction between models was less clear, but the model assuming selective reduction of k_n_ fit the data best, suggesting that SXkmer-VI-WI-DD inhibits primary nucleation.

### Binding analyses by SPR.

**Fig. 6.**
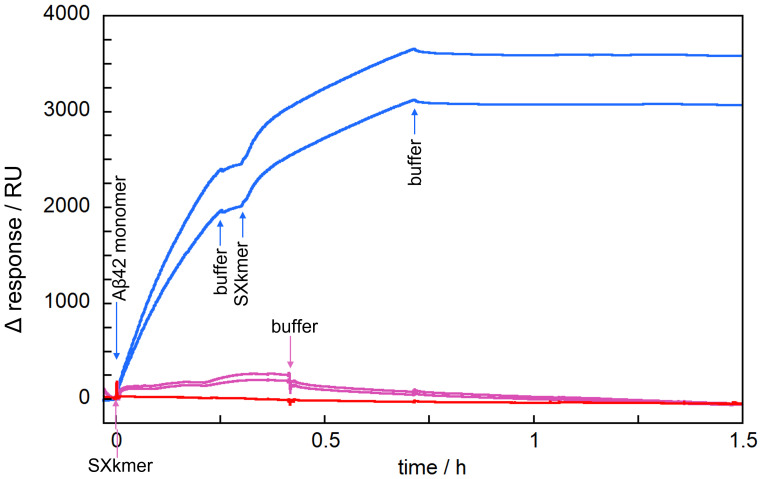
SPR analysis of SXkmer binding to oligomeric intermediates. The blue traces show examples of sensor grams (Δ response = observed response minus initial baseline) from the flow over immobilized Aβ42 fibrils (12,000 RU immobilized) with an initial injection of 150 µL 10 µM Aβ42 monomer, followed by an injection of 150 µL 1.0 µM SXkmer-YLTIRLM, and finally the buffer flow. The purple traces show examples of sensor grams from the injection of 250 µL 1.0 µM SXkmer-YLTIRLM over immobilized Aβ42 fibrils that had been equilibrated for 2 h past immobilization, followed by the buffer flow. The red traces show an example of a sensor gram from the injection of 250 µL 1.0 µM SXkmer-YLTIRLM over immobilized monomer (3,000 RU immobilized), followed by the buffer flow. Blank traces from flow cells with no coupled Aβ42 were subtracted from all traces before plotting.

The interaction between the SXkmer-YLTIRLM and Aβ42 was as monitored by SPR (Fig. 6) using sensor chip surfaces with immobilized monomers or fibrils. Little or no binding was observed in flow cells with immobilized Aβ42 monomers, and only small changes were seen in flow cells with immobilized and equilibrated Aβ42 fibrils. In contrast, a very significant increase in response was observed if Aβ42 monomers were injected over immobilized Aβ42 fibrils just prior to the injection of SXkmer-YLTIRLM (Fig. 6). While some of the injected monomers elongate the immobilized fibrils, a large fraction of the injected monomers adsorbs on the sides of fibrils, leading to the formation of fibril-associated oligomers (9). These oligomers may detach or convert to a fibrillar structure and detach (10), and our data imply that if SXkmer-YLTIRLM is injected while most of these oligomers remain on the fibrils, then there is a strong interaction between SXkmer-YLTIRLM and the oligomers on the fibrils, and the dissociation during the buffer flow is slow.

### Binding analyses in solution by FRET and MDS.

**Fig. 7.**
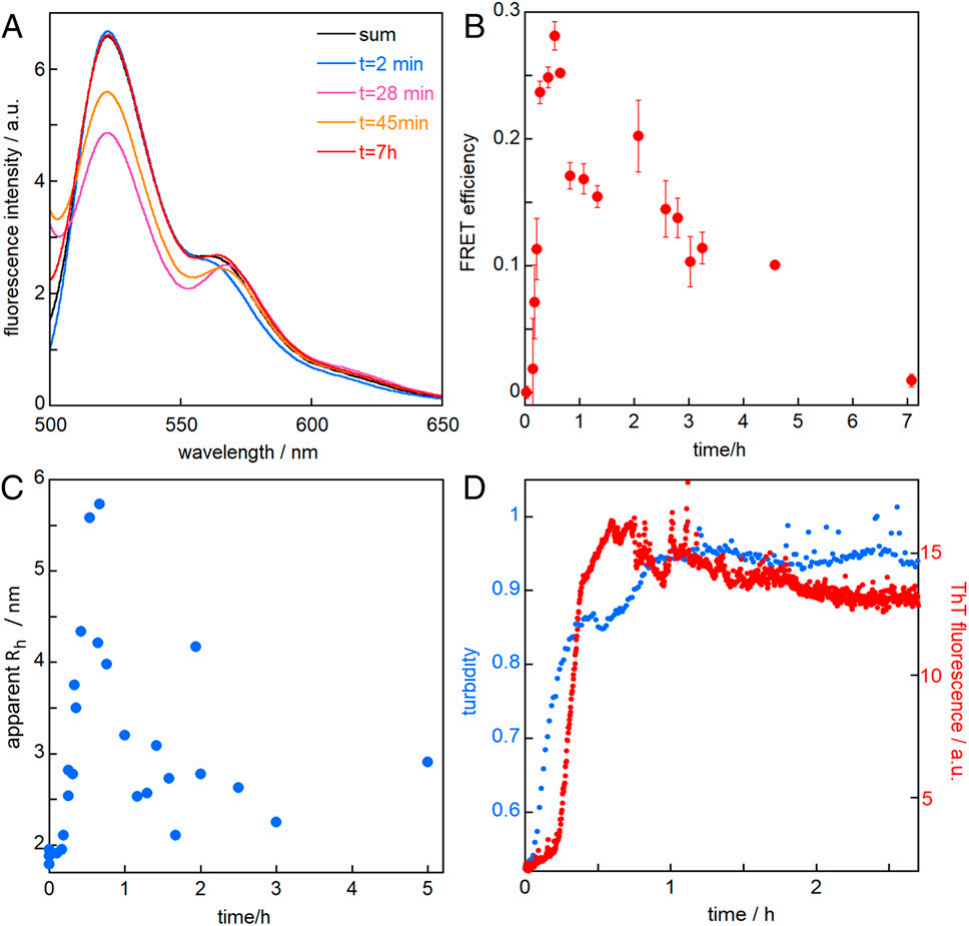
In-solution studies of SXkmer-YLTIRLM interaction with species withdrawn from an ongoing Aβ42 aggregation reaction. (*A*) Examples of fluorescence emission spectra for SXkmer-YLTIRLM-Alexa488 mixed with samples withdrawn from reactions of 9:1 Aβ42:Aβ42-Alexa555. The “sum” spectrum (black) is obtained by summing the spectrum of SXkmer-YLTIRLM-Alexa488 alone with that of Aβ42:Aβ42-Alexa555 alone. (*B*) FRET efficiency calculated from the fluorescence emission at 522 nm (i.e., from the donor SXkmer-YLTIRLM-Alexa488) shown as average and SD over three replicates. (*C*) Apparent hydrodynamic radius of Alexa-647–labeled species as a function of time of withdrawal from the ongoing Aβ42 reaction. (*D*) Examples of turbidity and ThT fluorescence traces from ongoing Aβ42 aggregation reactions. The samples followed by turbidity were used to acquire the data shown in panels *A*–*C*.

The interaction between the SXkmer-YLTIRLM and Aβ42, labeled with Alexa488 and Alexa555, respectively, was monitored using FRET (Fig. 7), in which case an interaction between SXkmer-YLTIRLM and Aβ42 would result in energy transfer between Alexa488 (emission at 522 nm) and Alexa555 and therefore reduced fluorescence emission at 522 nm. As shown in Fig. 7*B*, the fluorescence spectra recorded in the presence of monomers (2 min) or end-stage fibrils (7 h) are closely similar to the sum of spectra recorded separately for the two components, thus not revealing any interaction with monomers or fibrils. However, when SXkmer-YLTIRLM-Alexa488 was added to samples collected during an Aβ42 aggregation process (Fig. 7*D*), the fluorescence signal at 522 nm was attenuated, implying that SXkmer-YLTIRLM interacts with reaction intermediates rather than monomers or fibrils. The transfer efficiency, calculated from the signal intensity at 522 nm (Fig. 7*C*), displayed a sharp rise, a maximum around the midpoint of the fibril formation reaction, and a slower return to baseline. When instead fluorescence spectra were recorded continuously during an ongoing aggregation reaction in samples of 9 µM Aβ4, 1 µM Aβ42-Alexa555, and 0.1 µM SXkmer-YLTIRLM-Alexa488, the FRET effect developed over time but did not disappear over the time scale of the experiment (*SI Appendix*, Fig. S7), in line with the low dissociation rate inferred from the SPR data (Fig. 6).

The interaction between the SXkmer-YLTIRLM-Alexa647 and Aβ42 was also monitored using MDS. Samples of unlabeled Aβ42 were withdrawn at different time points from an ongoing reaction (Fig. 7*D*) and mixed with SXkmer-YLTIRLM-Alexa647, resulting in final total concentrations of 10 µM Aβ42 and 1 nM SXkmer-YLTIRLM-Alexa647. An interaction between SXkmer-YLTIRLM and Aβ42 would result in a reduced average diffusion rate and an increased apparent hydrodynamic radius (R_h_) of Alexa647-labeled species. The apparent R_h_ versus the reaction time (Fig. 7*C*) indeed displayed a short lag phase, a sharp rise, a maximum close to the midpoint of the fibril formation reaction, and a slower return to baseline.

## Discussion

The successful identification of an oligomer-specific binding protein that specifically inhibits the most toxic step along the Aβ peptide self-assembly process is a notable result of the current study. The SXkmer with loop–insertion YLTIRLM is a highly stable protein that selectively inhibits the secondary nucleation of Aβ42.

### Secondary nucleation inhibitors.

The selective inhibition of secondary nucleation can arise by more than one mechanism (10, 11). Inhibitors that bind along the sides of fibrils may block the catalytic sites for secondary nucleation. Molecules that bind to oligomeric structures formed on the fibril surface may prevent their conversion to a fibrillar structure. Examples in the first category are the Brichos chaperone domain, which completely stops the secondary nucleation of Aβ42 (12), and the antibody aducanumab, which may reduce the rate of secondary nucleation of Aβ42 up to threefold (14). These two examples represent secondary nucleation inhibitors in the form of human-derived proteins. Brichos domains are typically found in proteins with highly amyloidogenic segments, such as lung surfactant protein C, where the Brichos domain protects against amyloid formation in the lung (28). Aducanumab was isolated from an older cognitively normal adult individual with no signs of dementia, has been found to reduce brain amyloid and clinical symptoms (29, 30), and was recently approved by the US Food & Drug Administration (FDA) for AD treatment (31).

### Primary nucleation inhibitors.

The selective inhibition of primary nucleation can also arise by more than one mechanism (10, 32). Proteins that bind to monomer work through the law of mass action and reduce the primary nucleation rate by lowering the free concentration of monomers. Naturally occurring small molecules such as curcumin and resveratrol have been shown to bind to the N terminus (residues 5 to 20) of Aβ42 monomers, thereby interfering with primary nucleation (33). The affibody Z(Abeta3) is an example of a monomer binder, which suppresses primary nucleation by reducing the free monomer concentration (34). Molecules that bind to oligomeric structures, or form co-oligomers with amyloid proteins, may prevent their conversion to a fibrillar structure. The chaperone DNAJB6, which seems to inhibit both the primary and secondary nucleation of Aβ42 (15, 35), inhibits amyloid formation and increases the effective solubility of a range of aggregation-prone proteins (36, 37). It has been found to bind to oligomeric rather than monomeric forms of Aβ40 (38). DNAJB6 may thus interfere with oligomers both in solution and on the fibril surface and suppress their nucleation into a regular fibrillar structure (15, 35, 38, 39).

### Protein inhibitor design.

The present study was designed with a goal of developing proteins in the form of primary or secondary nucleation inhibitors. Previous efforts in this direction have employed an antibody design strategy to generate antibodies against different linear epitopes along the Aβ42 sequence (25) or phage–display selection of Aβ42 fibril-specific binders from a library of single-chain variable fragments (scFvs [13]). The latter approach has been extended and modified in two ways in the current work. First, two SXkmer libraries, built on the highly inert and stable scaffold S100G, were screened for binders to enable the derivation of more stable binders compared to scFvs. Second, we retrieved specific binders to oligomers as well as fibrils.

### Oligomer binders.

A major challenge in the field is the derivation of oligomer-specific binders. Although SXkmer-YLTIRLM was found in the selection versus immobilized Aβ42 monomers, it seems to bind to oligomers rather than monomers or fibrils. While an earlier study (13) used basic pH during bead coupling, the conditions used here for the coupling of purified monomers to silica nanoparticles (acidic pH) may have reduced the electrostatic repulsion between Aβ42 peptides and resulted in a mixture of immobilized monomers and oligomers. This enabled the monomer selection to retrieve both monomer binders and oligomer binders. SXkmer-YLTIRLM may belong to the latter category because the SPR, FRET, and MDS analyses failed to detect any interaction with monomers and found none or only a rather weak interaction with mature fibrils. However, if Aβ42 monomers were injected over Aβ42 fibrils right before the injection of SXkmer-YLTIRLM, then a strong increase in mass on the surface was observed by SPR, indicating an interaction between SXkmer-YLTIRLM and oligomeric structures on fibrils (Fig. 6). Likewise, both FRET and MDS revealed an interaction of SXkmer-YLTIRLM with intermediate species formed during the Aβ42 fibril formation process (Fig. 7). The time profiles of the FRET and MDS data resemble the oligomer concentration as a function of time (10), again making it highly likely that SXkmer-YLTIRLM interacts with oligomeric forms of Aβ42. The maximum FRET efficiency of 0.28 cannot be translated to an average interchromophore distance as this number depends on both the number of SXkmer-YLTIRM-488 bound per oligomer and the fraction of Aβ42-Alexa555 (in this case 10%) in the oligomers. The maximum apparent R_h_ is almost three times higher than for SXkmer-YLTIRM-647, corresponding to an almost 27 times higher apparent molecular weight of the diffusing species; however, because these are not necessarily globular and likely contain more than one SXkmer-YLTIRM-647 in addition to several Aβ42 peptides, we can only say that these species contain less than 50 Aβ42 monomers. The low off-rate observed by SPR (Fig. 6) and the continuous FRET measurements (*SI Appendix*, Fig. S7) suggest that the binding of SXkmer-YLTIRLM stabilizes oligomers and reduces their rate of dissociation or conversion to fibrillar species.

### Reverse engineering.

The current results may stimulate reverse engineering of a peptide or small molecule based on the loop or side sequence of selected peptide inhibitors from the SXkmer libraries as leads for future therapeutic modalities. Such reverse engineering is inspired by the fact that Aβ42 inhibitors are found also in the form of small molecules (40). One example is bexarotene, initially identified as a retinoid X receptor (RXR) agonist and approved by the FDA for the treatment of cutaneous T-cell lymphoma, which has been shown to also delay primary nucleation in Aβ42 aggregation and was used as a starting point for the design of new small molecule inhibitors (41, 42). The selection of the small molecules studied was based on data available on the atomic structures of the ligand binding domains and the chemical properties of known agonists and antagonists of retinoic acid receptors (RARs) and retinoid X receptors (RXRs), applying a form of structure-based drug discovery approach. Furthermore, based on molecular dynamics simulations, a negatively charged small molecule (ER) was predicted to sequester the intrinsically disordered Aβ42 in a monomeric and a pentameric state (43). Specifically, the ER small molecule inhibits the primary nucleation pathways.

The translation of peptide inhibitor sequences to small molecule compounds with the same bioactivity using structure-based design has been shown where nine small-molecule hit compounds of the heat shock 70kDa protein 5 were identified using a cascade in silico screening approach based on the binding modes of tetrapeptides derived from the peptide substrate or inhibitors of *E. coli* HSP70 (44). Here we show that the inhibitory effect of SXkmer-YLTIRLM is retained by the synthetic linear peptide corresponding to the seven–amino acid loop sequence, although the short peptide is less potent than the peptide as constrained in the SXkmer loop (Fig. 5). Truncating the YLTIRLM sequence from the N and C termini may reveal a minimum active sequence as a lead for conversion to a drug candidate. The influence of individual amino acids on the inhibition of secondary nucleation may also be assessed by systematically replacing each one with alternative amino acids in a focused phage–display library, and the role of conformational flexibility may be investigated by a stepwise reduction of the number of flanking glycine residues in the loop. This approach can be used to design a pharmacophore model in which functional groups crucial for activity are prepositioned (45).

The sequence alignment of SXkmer-YLTIRLM versus other sequences binding to the monomer/oligomer beads may provide hints toward further sequence optimizations. Our analysis reveals a pattern of alternating hydrophilic and hydrophobic residues (Fig. 3*E*) over the first six residues (YLTIRL). Such patterns have been linked with amyloid formation (46) and amyloid inhibition (25). Intriguingly, the hit sequence YLTIRLM obtained in the unbiased phage display selection procedure is similar to the rationally designed motif HETLTLR grafted in the single-domain antibody DesAb-Aβ3–9, which also binds the Aβ peptide (25). More specifically, the first five residues of our hit (YLTIR) are remarkably similar to the last five residues of the designed motif (TLTLR), and the nonidentical residues (Y vs. T and I vs. L) are both highly ranked in our sequence logo (Fig. 3*E*).

### Potential significance of the present findings.

In the past decade, there have been a number of significant advances in the identification and development of protein binder molecules that inhibit the different microscopic steps in the aggregation process of Aβ, through specific binding to monomers, fibrils, and oligomers (11–15, 35). The secondary nucleation of Aβ, believed to be the most toxic step in this aggregation process, is characterized by the formation of oligomeric species mediated by monomer binding to fibrils with subsequent conversion to fibrillar structures (8). Specific Aβ42 binders, a chaperone domain and an engineered antibody, have been shown to retard this conversion to fibrillar structures through specific binding to catalytic sites on the surface of fibrils (12, 14, 47). The development of engineered scaffold libraries and phage display has the potential to identify inhibitors that reduce the rate of secondary nucleation through binding to intermediate oligomeric structures on the fibril surface, thereby hindering their conversion to fibrils. The identification of immobilization conditions that promote the presentation of oligomeric species during phage display selections can promote the discovery of classes of therapeutic and diagnostic protein binders that interfere with the autocatalytic mechanism in protein aggregation and may also provide insights into the molecular processes underlying the autocatalytic mechanism.

## Conclusions

We have shown in this study that the creation of two diverse libraries based on a small and highly stable Ca^2+^-binding protein scaffold, combined with phage display and the selection of binders to monomers, oligomers, and fibrils of Aβ40 and Aβ42 proteins, enables the identification of highly specific pools of binding molecules. The structures of the binding interfaces of the side and loop libraries provide distinct and potentially complementary tools for the development of therapeutic and diagnostic entities. Notably, we have identified binders from these libraries that are specific not only to monomer and fibril species but also to oligomeric species through their effective presentation on silica nanoparticles under specific immobilization conditions. This selection of a binder protein specific to an oligomeric intermediate of Aβ42 from a library using phage display is an unprecedented finding and is likely to be a starting point in the selection of new oligomeric intermediate binders for AD therapy. This approach may be further developed and employed to study other amyloidogenic diseases where secondary nucleation is a key step in the catalysis of toxic oligomer formation.

## Methods

### Expression and purification of Aβ40 and Aβ42.

Aβ(M1-40) with the sequence MDAEFRHDSGYEVHHQKLVFFAEDVGSNKGAIIGLMVGGVV, here called Aβ40, and Aβ(M1-42) with the sequence MDAEFRHDSGYEVHHQKLVFFAEDVGSNKGAIIGLMVGGVVIA, here called Aβ42, were recombinantly expressed in *E. coli* from PetSac plasmids containing synthetic genes with *E. coli* optimized codons and purified from inclusion bodies using sonication, ion exchange, and two rounds of size exclusion chromatography, and the isolated monomers were stored as lyophilized aliquots as previously described (8, 14, 48). Aβ42-S8C was expressed and purified in the same way except that 1 mM DTT was included in all buffers up to the last SEC step, which served to isolate monomers in 20 mM phosphate buffer, pH 8.0, and to remove excess DTT (49).

### Fluorophore labeling of Aβ42.

The collected monomer fraction of Aβ42-S8C was lyophilized, dissolved in 50 µL 6 M GuHCl; 20 mM NaP, pH 8.0; and supplemented with 5 molar equivalents of Alexa555-maleimide from a 5 mM stock in DMSO. The solution was left at room temperature for 2 h, diluted in 1 mL 6 M GuHCl, and purified by SEC to isolate the labeled monomer and remove excess dye.

### Production of SXkmer phage–display libraries.

The name SXkmer alludes to the S100G scaffold and the size of 10 k (Xk). Each library was cloned in frame with gene III in the pIT2 phagemid vector for display at the N terminus of protein 3. The KM13 helper phage is used to rescue the SXkmer/pIII fusion that preferentially packages the single-stranded phagemid DNA in the presence of the phagemid wild-type M13 origin of replication. The use of the phagemid/helper phage also permits monovalent phage display for the selection of higher-affinity binders (50, 51). DNA codons were chosen to yield the following amino acid sequences of the displayed proteins:

$$\begin{array}{l} \text{SXKmer}-\text{loop}:\;\text{MKSPEELKRIFEKYAAKEGDPDQLSKDELKLLIQAEFPSLLKGM}\\ G\mathrm{GGXXXXXXXGGG}\text{STLDDLFQELDKDGDGEVSFEEFQVLVKKISQ}\\ \text{SXkmer}-\text{side}:\;\text{MKSPEELKRIFEKYAAKEGDPDQLS}XX\text{EL}XX\text{LI}XX\text{EFPSLLKGM}\\ \text{STLDDLFQELDKDGDGEVSFEEFQVLVKKISQ} \end{array}$$

where **X** denotes any amino acid except cysteine. The theoretical number of variants is 9·10^8^ for the loop library and 5·10^7^ for the loop library. The DNA synthesis and cloning into the pIT2 vector was purchased from Twist Biosciences. The libraries were received as glycerol stocks of *E. coli* Tg1. NGS sequencing of the libraries was executed by Twist. Aliquots of the glycerol stocks (0.5 mL) were added to 200 mL 2XTY medium and grown to an OD_600_ of 0.4, at which point helper phages were added. The cells were then grown overnight to release the phage-displayed libraries in the culture medium. The cultures were centrifuged, and the supernatant was mixed 4:1 with 20% PEG 6000, 2.5 M NaCl, and incubated in ice for 60 min. Phages were collected by sedimentation at 5,000 g for 30 min in a cold (4 °C) rotor. The pellet was collected again for 5 min, and all remaining PEG/NaCl was aspirated away before resuspending the phage in 4 mL PBS, pH 7.4.

### Preparation of monomers and fibrils.

Purified aliquots of Aβ40 and Aβ42 were separately dissolved in 1 mL 6M GuHCl and monomers were isolated using size exclusion chromatography on a 10/300 Superdex 75 column (GE Healthcare) in 20 mM sodium phosphate, 0.2 mM EDTA, pH 8.0 (Aβ42) or pH 7.4 (Aβ40), in low-binding tubes (Axygen) on ice. The peptide concentration was determined by the integrated absorbance of the collected fraction using ε_280_ = 1400 l mol^−1^ cm^−1^. Fibrils were prepared from the monomer solutions at quiescent condition at 37 °C in multiple wells of a PEG-ylated polystyrene 96-well plate (Corning 3881) with ThT in some wells. The ThT fluorescence was monitored through the bottom of the plate using a BMG Optima plate reader with excitation at 440 nM and emission at 480 nM until reaching the ThT plateau. Fibrils were collected from wells without ThT.

### Coupling to silica nanoparticles.

Silica nanoparticles were washed in 100 mM MES, pH 5.5, and activated by a mixture containing 0.2 M EDC and 50 mM NHS in water. Monomers or fibrils were diluted in 10 mM sodium acetate, pH 3.0, and incubated with the activated particles for 30 min, followed by blocking with 1 M ethanolamine and washing three times with 10 mM Tris/HCl; 150 mM NaCl; 100 µM CaCl_2_, pH 7.5; and 0.1% Tween20 (buffer A). After each step, the nanoparticles were pelleted by centrifugation.

### Phage display experiment for the selection of binding candidates.

Eight selections of binders were set up in parallel over three rounds in buffer A. The side and loop libraries (0.5 mL containing ca. 10^12^ phage particles) were separately incubated with the nanoparticle-conjugated Aβ40 or Aβ42 in the form of monomers or fibrils (Table 1). BSA was added at 2 mg/mL as a blocker of the particles before the third round of selection. After incubation with the libraries or amplified libraries for 1 h, the nanoparticles were washed 10 times with buffer A. After each incubation and washing step, the nanoparticles were pelleted by centrifugation. Phages were eluted through incubation with 100 µL 0.1 M HCl/glycine, pH 2.2, for 10 min, the nanoparticles were pelleted, and the supernatant was neutralized by adding 20 µL 1 M Tris, pH 9.1. Amplification in Tg1 *E. coli* was used to produce eight enriched libraries after each round according to the Tomlinson protocol.

### NGS.

Plasmids were prepared from cells pelleted from 2 mL ON cultures of Tg1 cells infected with the third-round eluates using a plasmid purification kit (Illustra). The purified plasmids were used as templates in PCR reactions with the following primers: S100Gfor (GGCCATGGCCAAATCTCC) and S100Grev (CCAGTTCCTGGAACAGGTC), yielding PCR products of 170 bp for side library members and 209 bp for loop library members. NGS of the PCR products, to yield 5 × 10^6^ reads using the same two primers as above, was purchased from Eurofins. NGS of the initial libraries was performed by Twist.

### Bioinformatics and sequence alignment.

The results of NGS were obtained as merged read files in the *fastq* format. Reads were converted to the sense direction (antisense reads were reverse-transcribed in silico). Reads containing undetermined N nucleotides, in-frame stop codons, or reads with any nucleotide with a phread quality score smaller than 21 (implying a confidence in the nucleotide identity < 99%), were discarded from further analysis. Similarly, we also discarded reads from the loop library that did not contain the expected nucleotide sequences in the regions flanking the randomized loops or from the side libraries with different nucleotides than those expected in the constant parts. *SI Appendix*, Fig. S1 shows the total and the retained number or reads for all sequenced screened libraries; the vast majority of the discarded reads were excluded by the in-frame stop codon and the phread quality criteria, as these were applied first.

Retained good-quality reads were then in silico translated into amino acid sequences and analyzed to provide files containing all sequences observed at least 100 times. For each library, sequences were ranked by their observed frequencies, and each of the first 500 sequences (or as many as found if fewer than 500) was then aligned against each of the other 499 sequences from the same selection and an alignment score was calculated using a scoring matrix with scores between 9 (identity) and 0 (no resemblance) (*SI Appendix*, Table S3). From these alignments were then generated lists of alignment above a chosen cutoff (here set to 30 for loop library selections and 27 for side library selections). The scores for 100% identity were 63 (loop) and 56 (side).

### Expression and purification of SXKmer proteins.

SXkmer proteins were expressed in *E. coli* from Pet3a plasmids containing synthetic genes with *E. coli*-optimized codons, with the desired mutations, and purified at pH 8.0 using sonication; boiling; two rounds of anion exchange in 10 mM Tris/HCl, 1 mM EDTA, and 10 mM Tris/HCl, 1 mM CaCl_2_, respectively; and size exclusion chromatography. Examples of chromatograms are shown in *SI Appendix*, Fig. S3. The isolated monomeric proteins were stored as frozen stocks.

### Fluorophore labeling of SXkmer-YLTIRLM.

SEC (on a G25 column) was used to isolate 100 µM SXkmer-YLTIRLM in 20 mM phosphate buffer, pH 8.0. The collected protein fraction was supplemented with 1.5 molar equivalents of Alexa488 carboxylate succinimidyl ester or Alexa647 carboxylate succinimidyl ester from 5 mM stock in DMSO. The solution was left at room temperature for 2 h and purified by SEC (on a Superdex75 column) to isolate the labeled protein and remove excess dye.

### SPR studies.

SPR was measured using a BIACORE 3000 instrument. Aβ42 monomers or fibrils were immobilized on CM3 sensor chips (Cytiva) using amine coupling. A fresh mixture of 0.05 M NHS and 0.2 M EDC was added to the sensor chip surface for activation, followed by washing and incubation with 1 µM Aβ42 monomers or sonicated fibrils in 10 mM NaAc, pH 3.0, and finally blocking by 1 M ethanolamine. One blank channel was prepared by omitting protein in the coupling step. The fibrils were further grown on the chip by an injection of monomer over the immobilized fibrils. The binding of SXkmer-YLTIRLM to monomers or fibrils was studied with or without the injection of monomers just prior, to enable oligomer formation on the immobilized fibrils.

### FRET with sample withdrawal.

Aβ42 wild-type and Aβ42-S8C-Alexa555, mixed at molar ratio 9:1, total peptide concentration 20 µM, were incubated in wells of a 96-well plate (Corning 3881 black polystyrene with PEG-ylated surface), and the reaction progress was monitored via the turbidity of the samples in a plate reader (BMG Clariostar). Samples withdrawn at discrete time points were mixed 1:1 (v:v) with 0.2 µM SXkmer-YLTIRLM-Alexa488, yielding final total concentrations of 10 µM Aβ42 and 0.1 µM SXkmer-YLTIRLM-Alexa488. Fluorescence emission spectra, 490 to 720 nm, 200 ms integration time, were recorded in a 3 × 3 mm quartz cuvette, total sample volume 80 µL, at 37 °C, using a ProbeDrum instrument (Probation Labs) with a 468 nm diode for excitation. The transfer efficiency was calculated from the signal intensity at 522 nm, I, as (I_0_-I)/I_0_, where I_0_ is the signal intensity in the absence of transfer.

Parallel samples in the 96-well plate, not used for the FRET study, were supplemented with 6 µM ThT, and the ThT fluorescence was monitored through the bottom of the plate as described above to allow comparison of the time dependencies of turbidity and ThT fluorescence.

### FRET measurements during an ongoing reaction.

Samples of 9 µM Aβ42 wt, 1 µM Aβ42-S8C-Alexa555, and 0.1 µM SXkmer-YLTIRLM-Alexa488 were placed in a 10 × 2 mm quartz cuvette, total sample volume 300 µL, sealed, and placed in a ProbeDrum instrument (Probation Labs) at 37 °C. The fluorescence emission spectrum of 490 to 720 nm was recorded at 1 min intervals for up to 60 h, 200 ms integration time, using a 468 nm diode for excitation. The static light scattering was measured in parallel for the same samples in the same cuvette at a right angle using a 637 m laser for excitation, 20 ms integration time. Continuous stirring at 60 rpm with a 2 mm stir bar was employed to reduce sedimentation out of the measurement window during the long time-course of these experiments.

### MDS.

The diffusion of SXkmer-YLTIRLM-Alexa647 alone or with Aβ42 withdrawn from an ongoing reaction was studied using a Fluidity One W serum instrument (Fluidics Inc. Cambridge, UK) using the intermediate flow rate, allowing for the diffusion of objects with a hydrodynamic radius ranging from 1.5 to 8 nm to be studied.

### Cryo–electron microscopy.

A controlled environment vitrification system was used to ensure a stable temperature and to avoid the loss of solution during sample preparation. Samples were prepared as thin liquid films (<300 nm thick) on glow-discharge treated lacey carbon film coated copper grids and plunged into liquid ethane at −180 °C. In this way, the original microstructures were preserved as component segmentation and rearrangement was avoided in addition to water crystallization as the samples were vitrified. Samples were stored under liquid N_2_ until measured and then transferred using an Oxford CT3500 cryoholder and its workstation into the electron microscope (Philips CM120 BioTWIN Cryo) equipped with a postcolumn energy filter (Gatan GIF100). An acceleration voltage of 120 kV was used, and images were recorded digitally with a CCD camera under low electron dose conditions.

## Supplementary Material

Supplementary File

## Data Availability

Data (sequence files, kinetic data) have been deposited in Dryad (https://doi.org/10.5061/dryad.4f4qrfjf6) (52).
