# Supplementary material for: An aggregation inhibitor specific to oligomeric intermediates of Aβ42 derived from phage display libraries of stable, small proteins
Source: Proc Natl Acad Sci U S A. 2022 May 17;119(21):e2121966119. doi: 10.1073/pnas.2121966119 (PMC9173773; doi:10.1073/pnas.2121966119)
Supplement: Supplementary File [file pnas.2121966119.sapp.pdf]

**An aggregation inhibitor specific to oligomeric intermediates of Aβ42 derived from phage display libraries of stable, small proteins**

Sara Linse[1], Pietro Sormanni[2], David O'Connell[3,4]

1. Lund University, Biochemistry and Structural Biology.
2. Cambridge University, Chemistry of Health, Yusuf Hamied Department of Chemistry
3. University College Dublin, School of Biomolecular and Biomedical Science
4. University College Dublin, BiOrbic, Bioeconomy SFI Research Centre

**Supplementary Information**

**Table S1. Codon Usage Tables for Side (A) and Loop (B) library construction**

**A.**

| Variant | 1 | 2 | 3 | 4 | 5 | 6 |
|---|---|---|---|---|---|---|
| ORF AA Position | 25 | 26 | 29 | 30 | 33 | 34 |
| ORF-AA | K | D | K | L | Q | A |
| ORF Codon | AAA | GAC | AAA | CTG | CAG | GCT |
| A | GCT | GCT | GCT | GCT | GCT | GCT |
| C | | | | | | |
| D | GAC | GAC | GAC | GAC | GAC | GAC |
| E | GAA | GAA | GAA | GAA | GAA | GAA |
| F | TTC | TTC | TTC | TTC | TTC | TTC |
| G | GGT | GGT | GGT | GGT | GGT | GGT |
| H | CAC | CAC | CAC | CAC | CAC | CAC |
| I | ATC | ATC | ATC | ATC | ATC | ATC |
| K | AAA | AAA | AAA | AAA | AAA | AAA |
| L | CTG | CTG | CTG | CTG | CTG | CTG |
| M | ATG | ATG | ATG | ATG | ATG | ATG |
| N | AAC | AAC | AAC | AAC | AAC | AAC |
| P | CCG | CCG | CCG | CCG | CCG | CCG |
| Q | CAG | CAG | CAG | CAG | CAG | CAG |
| R | CGT | CGT | CGT | CGT | CGT | CGT |
| S | TCT | TCT | TCT | TCT | TCT | TCT |
| T | ACC | ACC | ACC | ACC | ACC | ACC |
| V | GTT | GTT | GTT | GTT | GTT | GTT |
| W | TGG | TGG | TGG | TGG | TGG | TGG |
| Y | TAC | TAC | TAC | TAC | TAC | TAC |

**B.**

| Variant | 1 | 2 | 3 | 4 | 5 | 6 | 7 |
|---|---|---|---|---|---|---|---|
| **ORF AA Position** | 47 | 48 | 49 | 50 | 51 | 52 | 53 |
| **ORF-AA** | G | G | G | G | G | G | G |
| **ORF Codon** | GGT | GGT | GGT | GGT | GGT | GGT | GGT |
| **A** | GCT | GCT | GCT | GCT | GCT | GCT | GCT |
| **C** | | | | | | | |
| **D** | GAC | GAC | GAC | GAT | GAC | GAT | GAC |
| **E** | GAA | GAA | GAA | GAG | GAA | GAG | GAA |
| **F** | TTC | TTC | TTC | TTC | TTC | TTC | TTC |
| **G** | GGT | GGT | GGT | GGA | GGT | GGA | GGT |
| **H** | CAC | CAC | CAC | CAC | CAC | CAC | CAC |
| **I** | ATC | ATC | ATC | ATA | ATC | ATA | ATC |
| **K** | AAA | AAA | AAA | AAA | AAA | AAA | AAA |
| **L** | CTG | CTG | CTG | TTA | CTG | TTA | CTG |
| **M** | ATG | ATG | ATG | ATG | ATG | ATG | ATG |
| **N** | AAC | AAC | AAC | AAC | AAC | AAC | AAC |
| **P** | CCG | CCG | CCG | CCG | CCG | CCG | CCG |
| **Q** | CAG | CAG | CAG | CAG | CAG | CAG | CAG |
| **R** | CGT | CGT | CGT | CGT | CGT | CGT | CGT |
| **S** | TCT | TCT | TCT | TCT | TCT | TCT | TCT |
| **T** | ACC | ACC | ACC | ACC | ACC | ACC | ACC |
| **V** | GTT | GTT | GTT | GTC | GTT | GTC | GTT |
| **W** | TGG | TGG | TGG | TGG | TGG | TGG | TGG |
| **Y** | TAC | TAC | TAC | TAC | TAC | TAC | TAC |

**Table S2. The ten most frequent sequences obtained in each selection.**

| Side library | | | |
|---|---|---|---|
| **Aβ40 mono** | **Aβ40 fibril** | **Aβ42 mono** | **Aβ42 fibril** |
| VI--VI--DD | DQ--EG--HP | TA--RN--WA | WT--TV--VW |
| AP--LH--DE | SL--FP--DD | TH--PI--LS | PT--QI--HW |
| HK--SQ--WF | DA--NK--NP | MA--HA--GY | TP--AI--SY |
| PK--NR--FG | AY--PY--IP | MR--VE--HV | PP--QN--HF |
| YG--GY--HG | IW--NI--EL | NE--EH--NE | FL--DV--HA |
| EH--TL--LF | AI--YA--FK | GP--YA--IF | YY--TE--YV |
| YM--FI--AI | DY--PN--PP | HN--PD--QL | RQ--GF--LF |
| SI--RH--YE | EF--GP--EA | HS--EL--EY | KN--YW--VE |
| GD--HK--LY | GN--MQ--MM | VM--AN--GT | DS--KY--YW |
| FW--NI--VY | YY--DD--MP | HF--SR--NY | DD--FM--RM |
| **Loop library** | | | |
| **Aβ40 mono** | **Aβ40 fibril** | **Aβ42 mono** | **Aβ42 fibril** |
| VNIGLEY | IRQDAQA | QGKSVPA | EGVNEFF |
| LFVMTRM | RHRKPFE | YLTIRLM | IRWTVMM |
| HHYTVFM | GLDTRHD | ASNTYFS | GYRWWWV |
| ILALFFV | TLGKMHH | LIWGFKT | DRSNSPE |
| EDHREMD | KNMQMWV | PDPLDFD | RAHDASI |
| HPRSTAV | DYQPQGI | DLVSFYY | VHTKAAA |
| IMGYPLN | WPVGHAT | SWMALLV | DQWIEHV |
| FGVHEWV | MKQGPVY | SSWRGTT | NEMFVVW |
| PTDIWAW | FKRSWIF | HHQMTKS | DRQWYPA |
| PIHESEH | HITHNET | PWTVPVD | VHKFGHI |

**Table S3**. Sequence similarity scoring matrix with scores between 9 (identity) and 0 (no resemblance)

| | **A** | **C** | **D** | **E** | **F** | **G** | **H** | **I** | **K** | **L** | **M** | **N** | **P** | **Q** | **R** | **S** | **T** | **V** | **W** | **Y** |
|---|---|---|---|---|---|---|---|---|---|---|---|---|---|---|---|---|---|---|---|---|
| **A** | 9 | 0 | 0 | 0 | 1 | 3 | 0 | 1 | 0 | 1 | 1 | 0 | 1 | 0 | 0 | 3 | 2 | 3 | 0 | 0 |
| **C** | 0 | 9 | 0 | 0 | 0 | 0 | 0 | 0 | 0 | 0 | 0 | 0 | 0 | 0 | 0 | 5 | 3 | 0 | 0 | 0 |
| **D** | 0 | 0 | 9 | 7 | 0 | 0 | 0 | 0 | 1 | 0 | 0 | 5 | 0 | 1 | 1 | 2 | 3 | 0 | 0 | 0 |
| **E** | 0 | 0 | 7 | 9 | 0 | 0 | 0 | 0 | 1 | 0 | 0 | 1 | 0 | 5 | 1 | 1 | 1 | 0 | 0 | 0 |
| **F** | 1 | 0 | 0 | 0 | 9 | 0 | 3 | 6 | 0 | 6 | 3 | 0 | 3 | 0 | 0 | 0 | 0 | 5 | 3 | 3 |
| **G** | 3 | 0 | 0 | 0 | 0 | 9 | 0 | 0 | 0 | 0 | 0 | 0 | 0 | 0 | 0 | 0 | 0 | 0 | 0 | 0 |
| **H** | 0 | 0 | 0 | 0 | 3 | 0 | 9 | 0 | 0 | 0 | 0 | 0 | 0 | 3 | 6 | 0 | 0 | 0 | 3 | 3 |
| **I** | 1 | 0 | 0 | 0 | 6 | 0 | 0 | 9 | 0 | 8 | 5 | 0 | 3 | 0 | 0 | 0 | 0 | 5 | 2 | 2 |
| **K** | 0 | 0 | 1 | 1 | 0 | 0 | 0 | 0 | 9 | 0 | 0 | 1 | 0 | 3 | 6 | 0 | 0 | 0 | 0 | 1 |
| **L** | 1 | 0 | 0 | 0 | 6 | 0 | 0 | 8 | 0 | 9 | 5 | 0 | 3 | 0 | 0 | 0 | 0 | 5 | 2 | 2 |
| **M** | 1 | 0 | 0 | 0 | 3 | 0 | 0 | 5 | 0 | 5 | 9 | 0 | 3 | 1 | 0 | 0 | 0 | 2 | 2 | 2 |
| **N** | 0 | 0 | 5 | 1 | 0 | 0 | 0 | 0 | 1 | 0 | 0 | 9 | 0 | 5 | 0 | 2 | 2 | 0 | 0 | 0 |
| **P** | 1 | 0 | 0 | 0 | 3 | 0 | 0 | 3 | 0 | 3 | 3 | 0 | 9 | 0 | 0 | 0 | 0 | 2 | 0 | 0 |
| **Q** | 0 | 0 | 1 | 5 | 0 | 0 | 3 | 0 | 3 | 0 | 1 | 5 | 0 | 9 | 3 | 0 | 0 | 0 | 0 | 1 |
| **R** | 0 | 0 | 1 | 1 | 0 | 0 | 6 | 0 | 6 | 0 | 0 | 0 | 0 | 3 | 9 | 0 | 0 | 0 | 0 | 1 |
| **S** | 3 | 5 | 2 | 1 | 0 | 0 | 0 | 0 | 0 | 0 | 0 | 2 | 0 | 0 | 0 | 9 | 6 | 0 | 0 | 3 |
| **T** | 2 | 3 | 3 | 1 | 0 | 0 | 0 | 0 | 0 | 0 | 0 | 2 | 0 | 0 | 0 | 6 | 9 | 0 | 0 | 6 |
| **V** | 3 | 0 | 0 | 0 | 5 | 0 | 0 | 5 | 0 | 5 | 2 | 0 | 2 | 0 | 0 | 0 | 0 | 9 | 3 | 2 |
| **W** | 0 | 0 | 0 | 0 | 3 | 0 | 3 | 2 | 0 | 0 | 2 | 2 | 0 | 0 | 0 | 0 | 0 | 3 | 9 | 3 |
| **Y** | 0 | 0 | 0 | 0 | 3 | 0 | 3 | 2 | 1 | 2 | 2 | 0 | 0 | 1 | 1 | 3 | 6 | 2 | 3 | 9 |

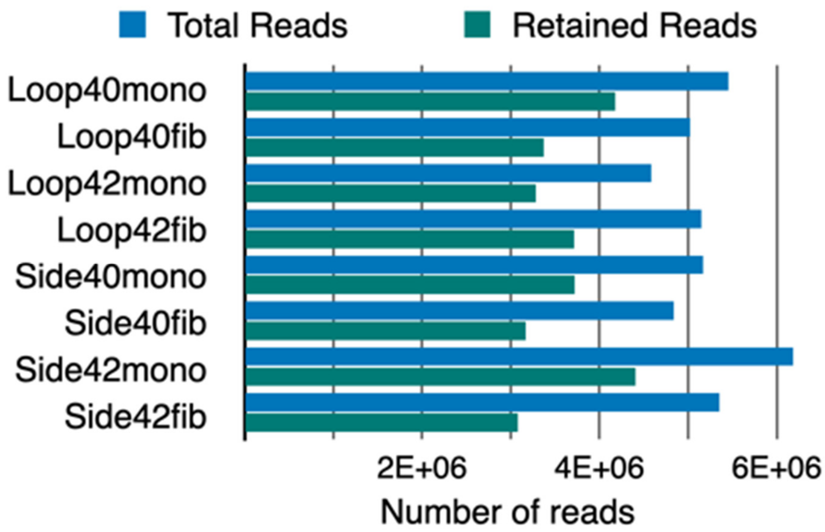


**Figure S1.** Number of reads obtained from the NGS reactions of the screened library. Some reads were discarded according to quality criteria (see Methods).

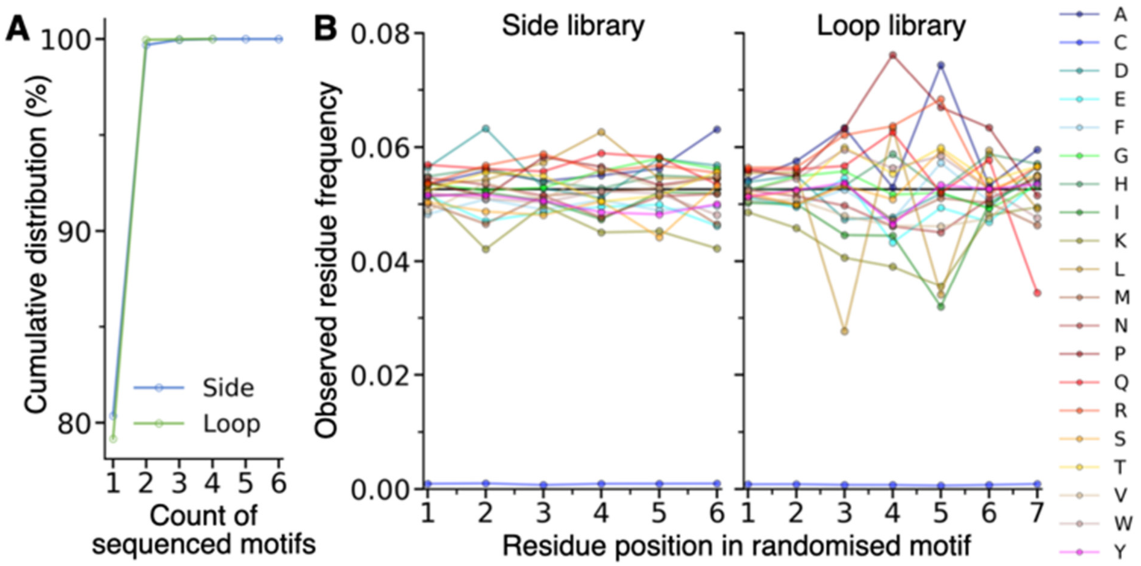


**Figure S2. NGS analysis of the naive libraries.** (**A**) cumulative distribution of the count of unique motifs among the NGS reads for the naïve libraries. The total number of reads was 818,985 and 637,264 for the side and loop library respectively, which corresponded to 681,556 and 527,409 unique motifs sampled. This cumulative plot shows that ~80% of these motifs were found only once, and > 99.9% were observed no more than twice in the sequencing reactions. (**B**) Observed amino acid frequency (y-axis) at each randomized position (x-axis) in the two libraries. For the side library, positions 1 to 6 correspond to the six randomized positions on the surface of S100G in the order they appear along the sequence (see Figure 1). The horizontal black line is the expected uniform frequency of 1/19. The observed frequency for cysteine residues (electric blue) is always < 0.001 and is likely arising from sequencing errors.

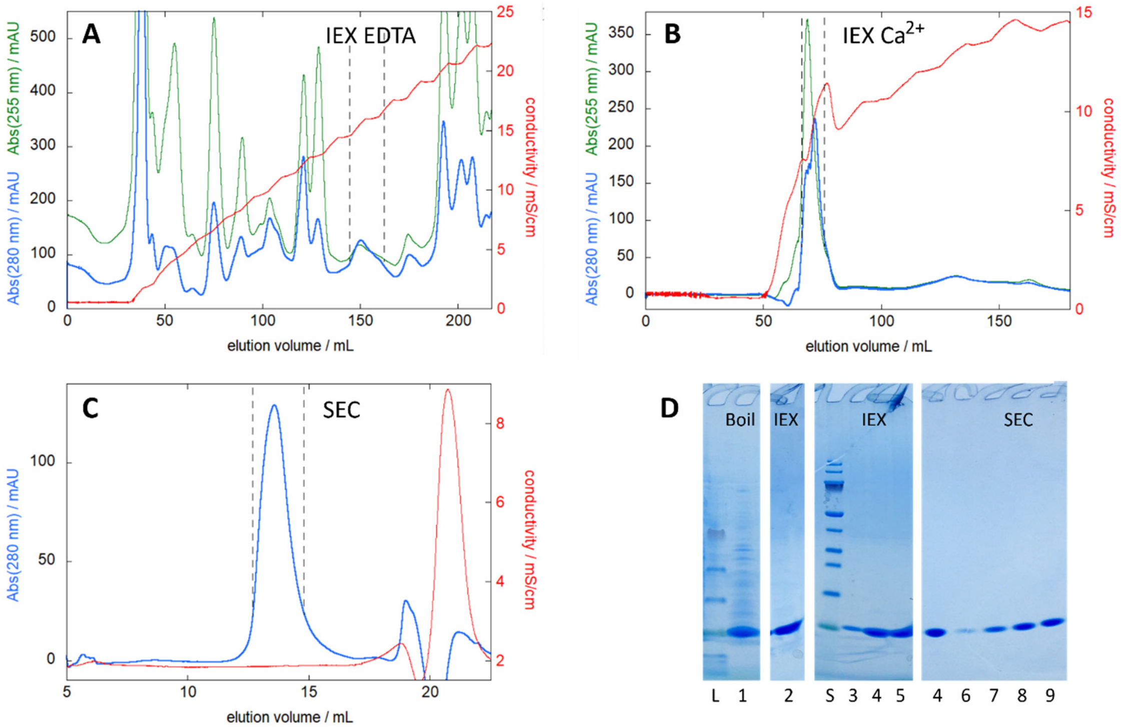


**Figure S3.** Purification of S100Gloop YLTIRLM. **A**. Elution of 5 mL QHP anion exchange column in 10 mM Tris/HCL, 1 mM EDTA with a linear salt gradient from 0 to 500 mM NaCl. The absorbance at 280 nm (blue) and 255 nm (green) and the conductivity (red) are shown. The vertical dashed lines indicate the fractions collected for next step. **B**. Elution of 5 mL QHP anion exchange column in 10 mM Tris/HCL, 1 mM $CaCl_2$ with salt gradient from 0 to 300 mM NaCl. The absorbance at 280 nm (blue) and 255 nm (green) and the conductivity (red) are shown. The vertical dashed lines indicate the fractions collected for next step. **C**. Elution of Superdex75 (10/300 mm) size exclusion column in 20 mM sodium phosphate, 0.2 mM EDTA, pH 8.0. The absorbance at 280 nm (blue) and the conductivity (red) are shown. The vertical dashed lines indicate the fractions collected for kinetics and binding analyses. D. SDS PAGE (4-20% gradient gel in 0.1 M Tris/tricine buffer) analysis of eluted fractions. L = low Mw standard (green band = 10 kDa), S = high Mw standard (green band = 10 kDa). Lane 1 = boiled cells supernatant, 2 = eluted fractions from first QHP column in EDTA, 3-5 = eluted fractions from second QHP column in $CaCl_2$ (nr 4 was applied to SEC column), 6-9 = eluted fractions from SEC.

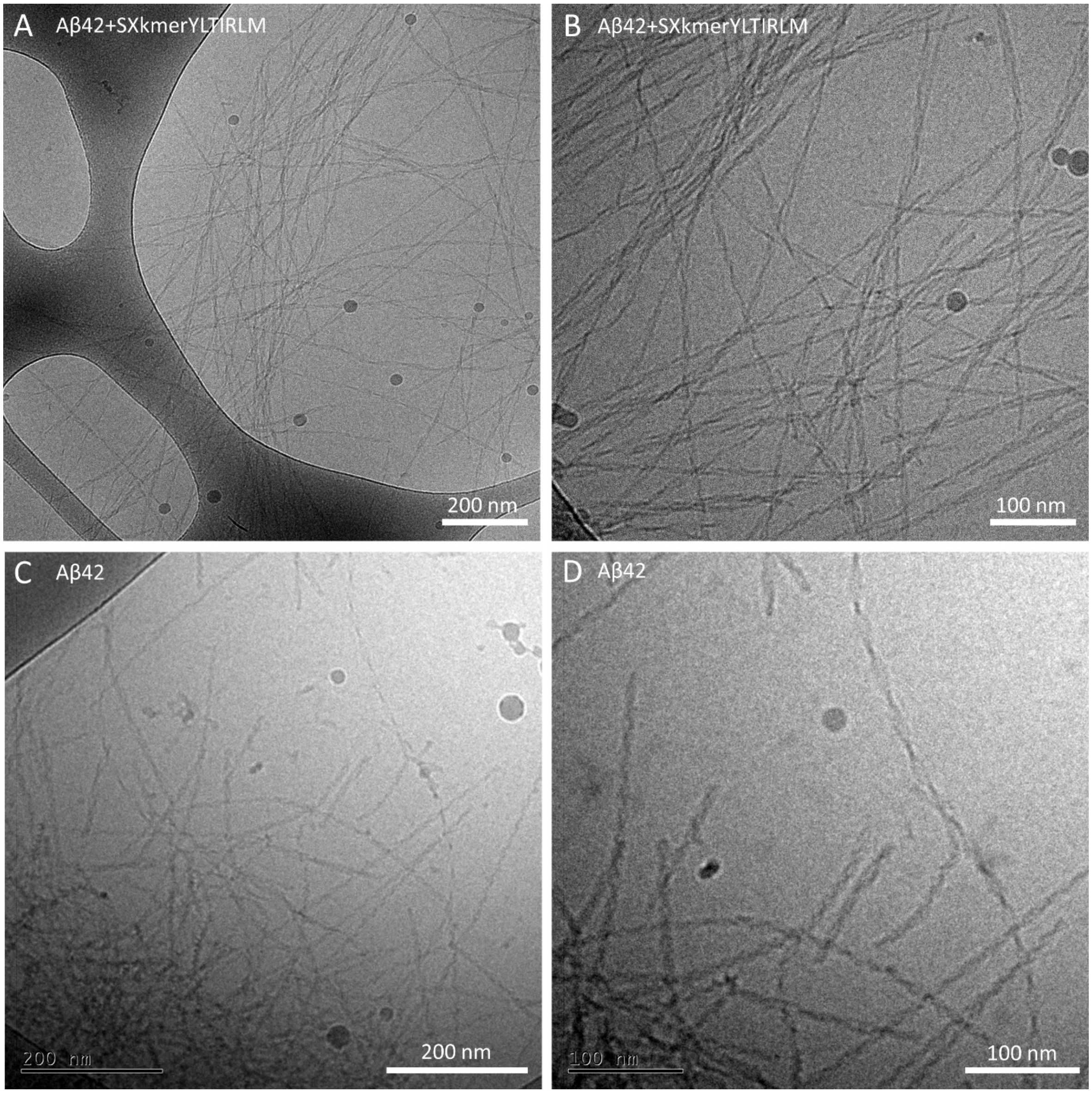


**Figure S4**. Additional cryo-TEM images of fibrils formed from Aβ42 in the presence of SXkmer-YLTIRLM (A,B) or Aβ42 alone (C,D).

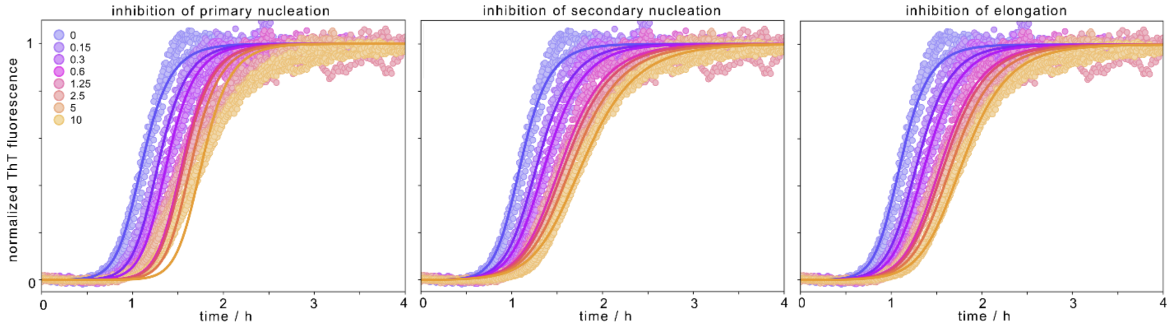


**Figure S5**. Effect of synthetic peptide YLTIRLM on Aβ42 aggregation kinetics. Aggregation kinetics of 3 μM Aβ42 in the absence and presence of YLTIRLM (separate colours for each concentration as indicated in μM) in 20 mM sodium phosphate, 0.2 mM EDTA, pH 8.0 monitored by ThT fluorescence. The data were fitted three times assuming selective reduction of the rate constant for primary nucleation (**left**), secondary nucleation (**middle**) and elongation (**right**).

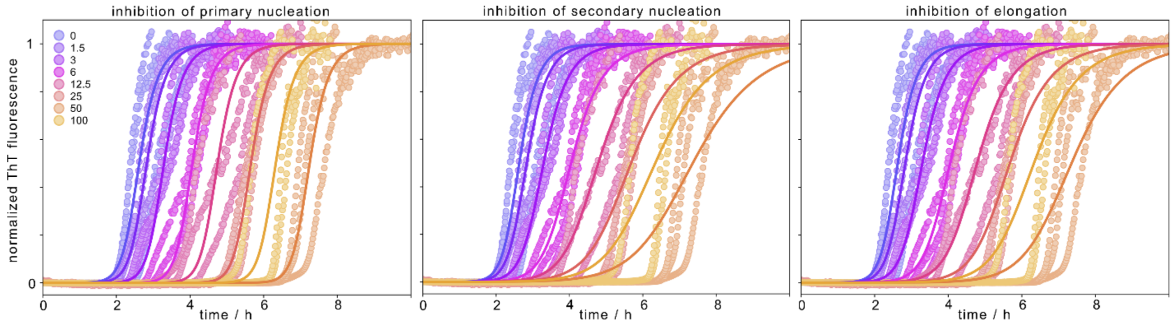


**Figure S6.** Aggregation kinetics of 15 μM Aβ40 in the absence (purple) and presence (purple to orange) of SXkmer-VI-WI-DD (the concentrations are indicated in the left panel in μM) in 10 mM Tris, 150 mM NaCl, 100 μM $CaCl_2$, pH 7.4 monitored by ThT fluorescence. The data were fitted three times assuming selective reduction of the rate constant for primary nucleation (**left**), secondary nucleation (**middle**) and elongation (**right**).

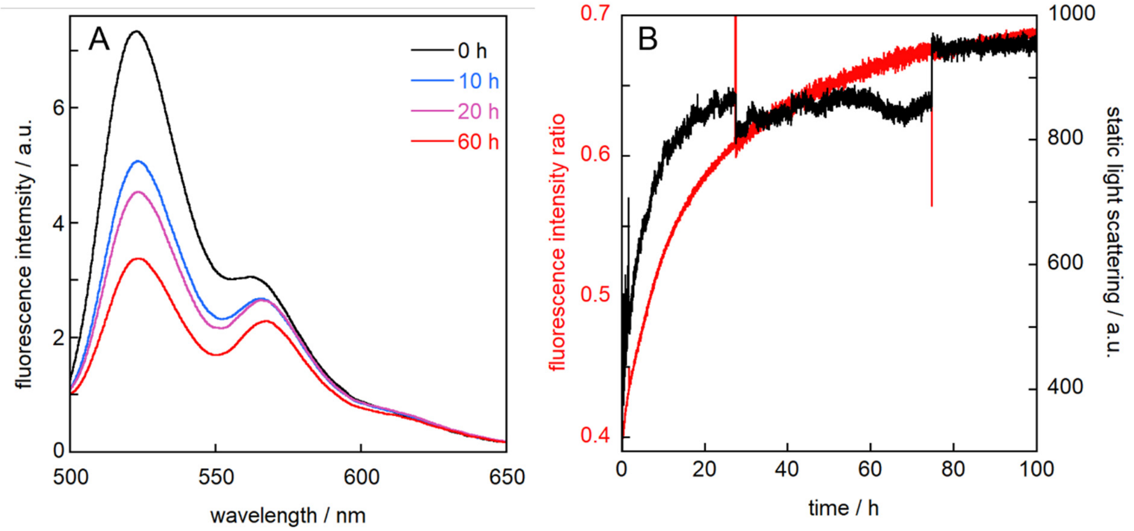


**Figure S7.** Continuous in-solution studies of SXkmer-YLTIRLM interaction with Aβ42 during an on-going Aβ42 aggregation reaction. A) Examples of fluorescence emission spectra for 0.1 μM SXkmer-YLTIRLM-Alexa488 mixed with 9 μM Aβ42 and 1 μM Aβ42-Alexa555, initially (0 h) and at three time points (10, 20 and 60 h) during an ongoing reaction. B) Relative fluorescence emission at 566 and 522 nm (red) and static light scattering (black) as a function of reaction time.
